# *SDHC* epi-mutation testing in gastrointestinal stromal tumours and related tumours in clinical practice

**DOI:** 10.1038/s41598-019-46124-9

**Published:** 2019-07-15

**Authors:** Ruth T. Casey, Rogier ten Hoopen, Eguzkine Ochoa, Benjamin G. Challis, James Whitworth, Philip S. Smith, Jose Ezequiel Martin, Graeme R. Clark, Fay Rodger, Mel Maranian, Kieren Allinson, Basetti Madhu, Thomas Roberts, Luis Campos, Joanne Anstee, Soo-Mi Park, Alison Marker, Colin Watts, Venkata R. Bulusu, Olivier T. Giger, Eamonn R. Maher

**Affiliations:** 1 Department of Medical Genetics, University of Cambridge and NIHR Cambridge Biomedical Research Centre and Cancer Research UK Cambridge Centre, Cambridge, CB2 OQQ United Kingdom; 2 Department of Endocrinology, Cambridge University NHS Foundation Trust, Cambridge, CB2 OQQ United Kingdom; 3 Department of Oncology, University of Cambridge, Addenbrooke’s Hospital, Cambridge, CB2 0QQ UK; 4 Department of Histopathology Cambridge University NHS Foundation Trust and Cancer Research UK Cambridge Centre, Cambridge, CB2 0QQ United Kingdom; 5 Cancer Research UK Cambridge Institute, University of Cambridge, Li Ka Shing Centre, Robinson Way, Cambridge, CB2 0RE UK; 6 Haematology Oncology Diagnostic Service (HODS), Cambridge University NHS Foundation Trust, Cambridge, CB2 OQQ United Kingdom; 7 Institute of Cancer and Genomic Sciences, College of Medical and Dental Sciences, University of Birmingham, Birmingham, B15 2TT UK; 8 Department of Medical Oncology, Cambridge University NHS Foundation Trust, Cambridge, CB2 OQQ United Kingdom; 9 Department of Pathology, University of Cambridge, Addenbrooke’s Hospital, Cambridge, CB2 0QQ UK

**Keywords:** Molecular medicine, Oncology

## Abstract

The enzyme succinate dehydrogenase (SDH) functions in the citric acid cycle and loss of function predisposes to the development of phaeochromocytoma/paraganglioma (PPGL), wild type gastrointestinal stromal tumour (wtGIST) and renal cell carcinoma. SDH-deficient tumours are most commonly associated with a germline SDH subunit gene (*SDHA*/*B*/*C*/*D*) mutation but can also be associated with epigenetic silencing of the *SDHC* gene. However, clinical diagnostic testing for an *SDHC* epimutation is not widely available. The objective of this study was to investigate the indications for and the optimum diagnostic pathways for the detection of *SDHC* epimutations in clinical practice. *SDHC* promoter methylation analysis of 32 paraffin embedded tumours (including 15 GIST and 17 PPGL) was performed using a pyrosequencing technique and correlated with *SDHC* gene expression. *SDHC* promoter methylation was identified in 6 (18.7%) tumours. All 6 *SDHC* epimutation cases presented with SDH deficient wtGIST and 3/6 cases had multiple primary tumours. No case of constitutional *SDHC* promoter hypermethylation was detected. Whole genome sequencing of germline DNA from three wtGIST cases with an *SDHC* epimutation, did not reveal any causative sequence anomalies. Herein, we recommend a diagnostic workflow for the detection of an *SDHC* epimutation in a service setting.

## Introduction

Loss of function of the succinate dehydrogenase (SDH) enzyme complex leads to intracellular accumulation of succinate as oxidative dehydrogenation of succinate to fumarate in the citric acid cycle is interrupted. Succinate can function as an ‘oncometabolite’ and drive tumourigenesis by competitively inhibiting 2-oxyglutarate dependent enzymes including prolyl hydroxylase and DNA and histone demethylase enzymes resulting in a pseudohypoxic transcriptional response^1^ and DNA and histone hypermethylation^2^.

Biallelic inactivation of one of the four SDH subunit genes (*SDHA, SDHB, SDHC, SDHD*) is the most common mechanism causing SDH deficient (dSDH) tumours. Germline genetic testing for germline *SDHx* mutations is now considered best practice for patients presenting with i) PPGL^3^, ii) wild type gastrointestinal stromal tumours (wtGIST)^4^ and iii) specific histopathological subtypes of renal cell carcinoma^5^. wtGIST are defined as GIST that are negative for *KIT* and *PDGFRA* somatic gene mutations and account for 15% of adult and 85% of paediatric GIST. Biallelic inactivation of any of the *SDHx* genes, most commonly results in destabilisation of the SDH enzyme complex, which can be detected by loss of staining for the SDHB protein on IH^6^ and therefore wtGIST can be further classified based on the loss or preservation of SDHB protein expression on immunohistochemistry as a surrogate marker for loss of function of the SDH complex. Importantly, SDH deficient wtGIST (dSDH wtGIST) account for approximately 7–10% of all GIST^4,7^.

Identification of a germline pathogenic variant in *SDHB* informs a higher risk of a malignant PPGL^2^ and detection of a germline *SDHx* mutation facilitates personalised surveillance, family screening and potentially the choice of therapy for metastatic disease^1,2^. In addition to testing for germline *SDHx* variants, immunostaining for SDHB and SDHA is a valuable approach for identifying dSDH tumours^6^.

It is now recognized that in a subset of dSDH tumours, SDH inactivation results from promoter hypermethylation and epigenetic silencing of the *SDHC* gene^2,6–10^. *SDHC* promoter hypermethylation has been most frequently found in dSDH-wtGIST^8–13^ with up to a third of all of cases having *SDHC* promoter methylation^2^. Distinguishing dSDH tumours with germline *SDHx* mutations from those with *SDHC* hypermethylation only is beneficial because i) the relatives of patients with a germline *SDHx* mutation are at increased tumour risk and ii) an *SDHC* epimutation is potentially reversible (clinical trials have been initiated to investigate demethylating agents in such cases (ClinicalTrials.gov Identifier: NCT03165721)).

*SDHC* epimutations appear to be unique to specific tumour types (e.g wtGIST and PPGL)^8^ but further study is required to determine whether *SDHC* epimutations might occur in tumours with an associated hypermethylation phenotype other than SDH deficient wt GIST and PPGL. *IDH1* mutant gliomas have previously been associated with a global hypermethylation phenotype due to inhibition of alpha ketoglutarate dependent de-methylation enzymes^14^ and therefore *IDH1*mutant gliomas are a useful tumour type to test the hypothesis that *SDHC* promoter hypermethylation is unique to specific tumour types.

Despite the implications for patient management and family testing and screening, diagnostic testing for *SDHC* epimutations has not been adopted as routine clinical practice because the indications for testing and a suitable methodology for a clinical service laboratory have not been well defined^8^. The aims of this study were; i) to investigate a pyrosequencing-based assay for the diagnosis of *SDHC* promoter methylation and ii) to determine the role for *SDHC* epimutation testing in a clinical diagnostic pathway using pooled data from this study and available literature.

## Methods

### Clinical sample collection

Cases were ascertained from the Neuroendocrine Tumour, the National Pediatric and Adult wild type GIST (PAWS GIST UK) and clinical genetics clinics at Cambridge University Hospital NHS Foundation Trust. Details of clinical phenotype, family history and germline molecular testing results were collated from patient records.

### Study design

All cases of identified PPGL wtGIST, for whom formalin fixed paraffin embedded (FFPE) tumour blocks were available, were considered for inclusion in the study. All participants (and or legal guardians) gave written informed consent. 32 cases (15 wtGIST and 17 PPGL) were included in the analysis. For each case studied, DNA was extracted from FFPE tumour tissue and adjacent normal tissue (31/32 cases) and blood when available (21/32 cases). mRNA was extracted from FFPE tumour tissue and adjacent normal FFPE tissue. SDHB immunohistochemistry (IH) was performed on all 32 samples. Tumour samples with evidence of SDHB preservation on SDHB IH were included in *SDHC* promoter methylation analysis in order to confirm if SDHB IH was a sensitive triaging test for the diagnosis of an *SDHC* epimutation.

Methylation analysis was performed on DNA extracted from FFPE tumour and matched normal tissue/blood. *SDHC* expression analysis was performed on RNA extracted from FFPE tumour and matched normal tissue and finally sequencing of tumour DNA was performed to identify somatic *SDHx* mutations.

A further 17 *IDH1* mutant glioma samples (anonymised tumour DNA from consented patients provided by Professor Colin Watts) were included in the study.

### Germline and tumour genetic sequencing

#### Clinical germline DNA sequencing

DNA was extracted from peripheral blood samples according to standard protocols. Next generation sequencing of a clinical gene panel including; *SDHA*, *SDHB*, *SDHC*, *SDHD*, *KIT, PDGFRA* and *NF1* (for GIST) and *SDHA*, *SDHB*, *SDHC*, *SDHD, SDHAF2*, *MAX*, *TMEM127*, *VH*L, *RET*, *FH* for (PPGL) was performed by the laboratory staff at Cambridge University Hospital NHS Foundation Trust or Birmingham Women’s and Children’s Hospital NHS Trust using the TrusightOne or Trusight Cancer sequencing panels (Illumina Inc., UK).

An average coverage depth of >20 fold was achieved for 98% of the regions sequenced. All detected variants were confirmed by Sanger sequencing. Whole exon deletions and duplications and large rearrangements are not detected using this method and multiple ligation probe analysis (MLPA) was performed for *VHL*, *SDHB*, *SDHC* and *SDHD*.

#### Tumour DNA sequencing using a custom gene panel

Tumour sequencing was performed on those cases with sufficient DNA quantity following methylation analysis (27/32 cases of PPGL/GIST and 17 gliomas) by the staff at the Stratified Medicine Core Laboratory within the Department of Medical Genetics, Cambridge University. Sequencing was performed using a custom panel based on the Ion AmpliSeq™ 142 Cancer Hotspot Panel v2 (catalogue number 4475346).

Variant filtering was performed on variant calling files (VCF). Variants were removed if the variant allele frequency was <10% or the minor allele frequency (MAF) greater than 0.1% in EVS6500 and/or 1000 genome project (www.internationalgenome.org). Synonymous variants were removed as presumed not to be pathogenic. Those variants that had coverage of less than two standard deviations below the mean coverage were also removed.

#### Data extracted from whole genome sequencing

Whole genome sequencing (WGS) was performed on germline DNA from three cases as part of the NIHR Rare Disease Bioresource project and sequencing data from two of the three patients was included in a recent publication^15^. Data was filtered to include data in the regions of interest: the *SDHC* promoter region and five genes involved in DNA methylation maintenance and regulation: *TET1*, *TET2*, *TET3*, *DNMT3A* and *DNMT3B*.

The variants were annotated with variant effect predictor and filtered on i) minor allele frequency of <0.1 or absent in 1000 genome project (www.internationalgenome.org) and UK10K (https://www.uk10k.org), ii) consequence including; truncating, missense, splice site and in frame deletion and insertion variants and iii) quality including; a read depth of >10 and variant allele frequency of >0.3. All filtered variants were then individually interrogated and assigned pathogenicity based on American College of Medical Genetics and Genomics (ACMG) criteria.

A comparison of variant allele frequencies in our samples compared to a control group with low neoplastic risk within the bio resource project (NIHR rare disease controls, n = 4053), was also performed and calculated using a Fishers exact test and corrected for a false discovery rate using the Benjamini-Hochberg procedure. Finally, cases were evaluated for structural variants (SV) including copy number variation, using the SV calling tools; Canvas and/or Manta^16,17^.

### Tissue dissection for DNA and RNA isolation

Pre-selected paraffin blocks containing tumour and adjacent normal tissue were used for nucleic acid extraction. Tumour tissue and normal tissue suitable for DNA isolation was identified by an experienced molecular histopathologist (OG). Tumour cell content in the tumour enriched areas ranged between 50–80%. Normal tissue used as control was histologically confirmed to be tumour free. 6–10 µm thick FFPE sections were mounted on glass slides. Tumour and normal tissue were scraped of the slides barring a security margin between tumour and normal of 2 mm.

### Bisulfite modification

The assay was proven to work reliably with 10 ng input DNA, however 500 ng of DNA was used as a standard for bisulfite modification with the Zymo Research EZ DNA Methylation kit (D5001) according to the manufacturer’s instructions. Bisulfite converted DNA was eluted from the spin colums with 50ul of elution buffer and directly processed for PCR or frozen at −20 °C. Complete bisulfite modification was monitored by an internal bisulfite control position after 5 consecutive cytosines in the genomic sequence in the pyrosequencing assay.

### Polymerase chain reaction and pyrosequencing

CpG27 was chosen over CpG17 as the CpG27 island was located proximal to the transcription start site for the *SDHC* gene. A 198 bp sized PCR amplicon in the CpG27 island located in the *SDHC* promoter region of the *SDHC* gene was amplified from 50 ng of CT bisulfite converted DNA with 375 nM of forward primer (GAAAATAATTAGTAAATTAGTTAGGTAG) and 187.5 nM of biotinylated reverse primer (ACTAAAATCACCTCAACAACAAC) with the Qiagen PyroMark kit (Qiagen 978703). The PCR conditions were 7 min at 95 °C, followed by 20 sec at 95 °C, 30 sec at 53 °C, and 20 sec at 72 °C for 42 cycles, and an end incubation at 72 °C for 5 min. The resulting PCR amplicon was quality assessed for purity and yield on a 2% agarose gel. A nested sequencing primer (GTTATATGATATTTTTAATTT) at a concentration of 500 nM was used to analyse 12 CpGs in 10ul of the sample on the Qiagen Q24 pyrosequencer. Fully methylated and unmethylated human control DNA that had been treated with bisulfite were used as controls on each pyrosequencing run.

Ten percent of the bisulfite conversion eluate (approximately 50 ng) was used as a PCR template. The lower detection limit of the assay was 10% eluate of 10 ng input DNA for bisulfite conversion (approximately 1 ng) for fresh frozen and DNA isolated from FFPE. Methylation percentage differences of 25% were reliably detectable for 10 ng and 50 ng of template bisulfite converted DNA.

### Development of a clinical diagnostic assay for SDHC methylation

In order to facilitate the translation of *SDHC* promoter methylation analysis into clinical practice we set out to develop an assay using technology that is available in a service setting and that would provide robust results on DNA extracted from FFPE. Tumours from 32 patients with wtGIST^15^ and PPGL^17^ and a further 17 glioma tumour samples were studied.

**Additional methods in supplementary data**: **(**i) Tumour DNA extraction, (ii) Analysis of TCGA tumour set, (iii) RNA extraction, (iv) cDNA synthesis, (v) Expression Analysis with quantitative RT PCR, (vi) Statistical analysis.

All methods were performed in accordance with the relevant guidelines and recommendations.

### Ethical approval and consent to participate

All participants gave written informed consent for study participation and publication and the study was approved by Cambridge South Research Ethics Committee (REC Reference Number: CA/5175).

## Results

### Genotype and clinical phenotype of patient cohort

#### wtGIST and PPGL cases

The mean age of tumour diagnosis was 36.6 years (range 15–71, SD 18.8). The fifteen cases of wtGIST included 10 cases of dSDH-wtGIST and 5 cases of SDH preserved wtGIST, as defined by loss or preservation respectively of SDHB protein expression on immunohistochemistry (Table 1). The 17 PPGL cases included 13 SDH preserved PPGL, 3 dSDH-PPGL and 1 PPGL with an equivocal SDHB result (diffusely weak SDHB expression) (case # 026) (Table 2). Thirteen participants were male, 19 female and nine cases had metastatic disease (Tables 1 and 2). Five patients had a clinical history of multiple primary tumours (Tables 1 and 2).

**Table 1:** Clinical and molecular profile of wtGIST.

Case	Age	Sex	Germline variants	Phenotype	Mean tumour methylation index (MI%)	SDHB IH
001	23	F	Negative*	Metastatic wtGIST	73%	Loss
002	15	F	Negative*	Metastatic wtGIST	45%	Loss
003	21	F	Negative*	Metastatic wtGIST	68%	Loss
004	24	F	*SDHC* c.380A>G, p.His127Arg	wtGIST + Oesophageal leiomyoma + Pulmonary chondroma	38%	Loss
019	16	F	*SDHA* c.91C>T p.Arg31Ter	Metastatic wtGIST	3%	Loss
020	37	M	*SDHB* c.137G>A p.Arg46Gln	Metastatic wtGIST + Carotid PGL	1%	Loss
021	21	F	*SDHD* c.34G>A (p.Gly12Ser) (benign polymorphism)**	Metastatic wtGIST + Thoracic PGL	49%	Loss
022	27	F	*SDHC* c.148C>T p.Arg50Cys*	wtGIST + Abdominal PGL	32%	Loss
023	29	F	Negative	wtGIST	7%	Preserved
024	36	F	*NF1* c.4421delG p.Tyr794Ter	wtGIST	4%	Preserved
027	22	F	Negative	wtGIST	1%	Preserved
028	24	F	*SDHA* c.1909-2A>G	Metastatic wtGIST	2%	Loss
030	30	M	Negative	wtGIST	1%	Preserved
031	57	M	Negative	wtGIST	1%	Preserved
032	67	M	*SDHD* c.296delT, p.Leu99Profs*36	wtGIST	2.5%	Loss

*MLPA performed.

**Benign polymorphism.

**Table 2:** Clinical and molecular profile of PPGL study participants.

Case	Age	Sex	Germline variants	Phenotype	Mean tumour methylation index (MI%)	SDHB IH
005	22	F	*SDHB* c.380G>T p.Ile127Ser	Abdominal PGL	5.5%	Loss
006	27	M	*SDHB* c.302G>A p.Cys101Tyr	Abdominal PGL + Carotid PGL	1%	Loss
007	15	M	Negative*	Abdominal PGL	2%	Loss
008	21	M	Negative*	PC	2%	Preserved
009	40	F	Negative*	Metastatic PC	1%	Preserved
010	38	F	*NF1* c.1318C>T p.Arg440Ter	PC	1%	Preserved
011	78	F	Negative*	PC	1%	Preserved
012	38	F	*RET* c.1900T>A p.Cys634Ser	PC	1%	Preserved
013	30	M	Negative*	PC	1%	Preserved
014	62	M	Negative*	PC	2%	Preserved
015	37	F	*RET* c.1900T>A p.Cys634Ser	PC	1%	Preserved
016	52	M	Negative*	PC	2%	Preserved
017	78	M	Negative*	PC	6%	Preserved
018	45	M	Negative*	Abdominal PGL	5%	Preserved
025	72	M	Negative*	PC	1%	Preserved
026	25	F	*VHL* c.499C>G p.Arg167Gly	PC	1%	Equivocal
029	27	F	Negative*	Metastatic PC	2%	Preserved

*MLPA performed.

A likely pathogenic or pathogenic germline variant was identified in 12/32 patients (37.5%; 6/15 GIST and 6/17 PPGL). No CNV was identified by MLPA testing in the cohort.

### Methylation analysis by pyrosequencing of tumour DNA from wtGIST and PPGL cohort

**Figure 1:**
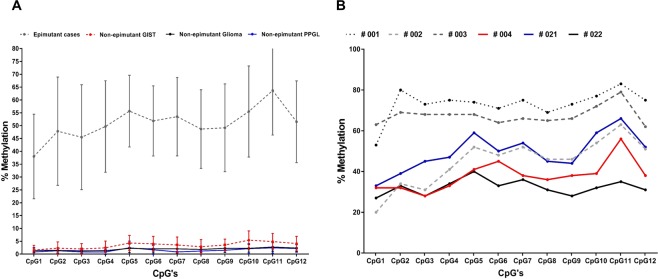
Figure (**A**) illustrates the distribution of methylation across the 12 individual CpG’s for the six cases demonstrated to have *SDHC* promoter methylation (epimutant cases), and the wt GIST, glioma and PPGL cases with no *SDHC* epimutation. Figure (**B**) demonstrates the methylation levels across the 12 individual CpG’s for the six epimutated cases (#001, #002, #003, #004, ##021, #022).

The % methylation at each of the 12 CpG’s in CpG island 27(CpG27) in the promoter region of *SDHC* was tested. The percentage methylation ranged between 1% and 73% but was highly correlated within an individual tumour sample with no significant variability detected across individual CpGs (p = 0.08) (see Fig. 1). A mean % methylation index (MI = % of methylated CpGs) of 2.2% (±SD 1.98) across 12 CpG’s, was detected in all but 6 tumour samples (Table S1). The mean MI in these six tumours was 50.8% (±SD 16.4) (Fig. 1B) (cases: #001, #002, #003, #004, #021, #022).

All cases identified as having an *SDHC* epimutation in this study had a dSDH wtGIST as the presenting phenotype. Comparing 6 tumours with evidence of *SDHC* hypermethylation to those with low methylation revealed statistically significant associations with wtGIST (6/15 versus 0/17 PPGL; P = 0.005), female sex (6/19 versus 0/13 males; P = 0.02); metastatic disease (5/6 versus 5/26 (P = 0.035), younger age at diagnosis (mean age 24 years versus mean age 39.2 years) (P = 0.0002) and multiple primary tumours (3/6 versus 2/26, P = 0.03). No significant association was found for the presence of a germline pathogenic *SDHx* variant (P = 0.2).

### Methylation analysis by pyrosequencing of blood and adjacent normal tissue DNA from wtGIST and PPGL cohort

The purpose of this analysis was to further investigate whether *SDHC* promoter hypermethylation is a constitutional, mosaic or somatic event.

**Figure 2:**
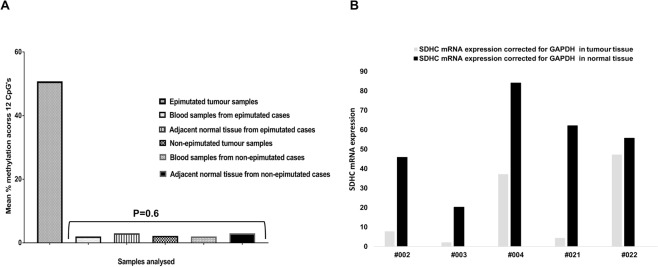
Figure (**A**) shows the difference in the mean % methylation of the *SDHC* promoter locus across 12 CpG’s in the tumour of the six hypermethylated cases and tumours of the non-epimutant cases and blood DNA and normal tissue of cases with and without an identified *SDHC* epimutation. Figure (**B**) shows reduced *SDHC* expression in the tumour versus normal tissue of 5/6 cases with an identified *SDHC* epimutation.

Pyrosequencing of blood DNA was performed on 22/32 (69%) wtGIST and PGL cases and matched normal tissue for 31/32 cases (97%). No evidence of *SDHC* promoter hypermethylation was detected in blood or normal tissue (MI <10% in all samples) including the 6 samples with tumour *SDHC* hypermethylation. No statistically significant difference was identified between the mean MI in blood DNA or adjacent normal tissue for those cases identified as having tumour hypermethylation compared with those cases without tumour methylation (p = 0.6) (Fig. 2A).

As expected, a significant difference was noted for the MI in the tumour compared to the adjacent normal tissue for the 5 hypermethylated tumour cases for which adjacent normal tissue was available for testing (p = 0 .003) (Fig. 2A). ROC curve analysis (see Supplementary Data and statistical methods) demonstrated that a methylation of >8.5% separated the cases with an identified epimutation and silencing of *SDHC* from those without (AUC 1.0, p = < 0.0001).

### Analysis of SDHC gene expression in tumour tissue from wtGIST and PPGL cohort

To determine whether *SDHC* promoter methylation was associated with transcriptional silencing, analysis of *SDHC* mRNA in both tumour tissue and adjacent normal tissue was performed in 31/32 cases. In 5/5 tumour samples with *SDHC* hypermethylation the mean fold difference was −6.41(SD 5.4) (Fig. 2B) compared to 1.41 (SD 4.41) in 26 tumours without *SDHC* hypermethylation (P = 0.0002) (Figure S1).

### Tumour sequencing and additional functional analysis for SDH deficiency in the hypermethylated cases

**Figure 3:**
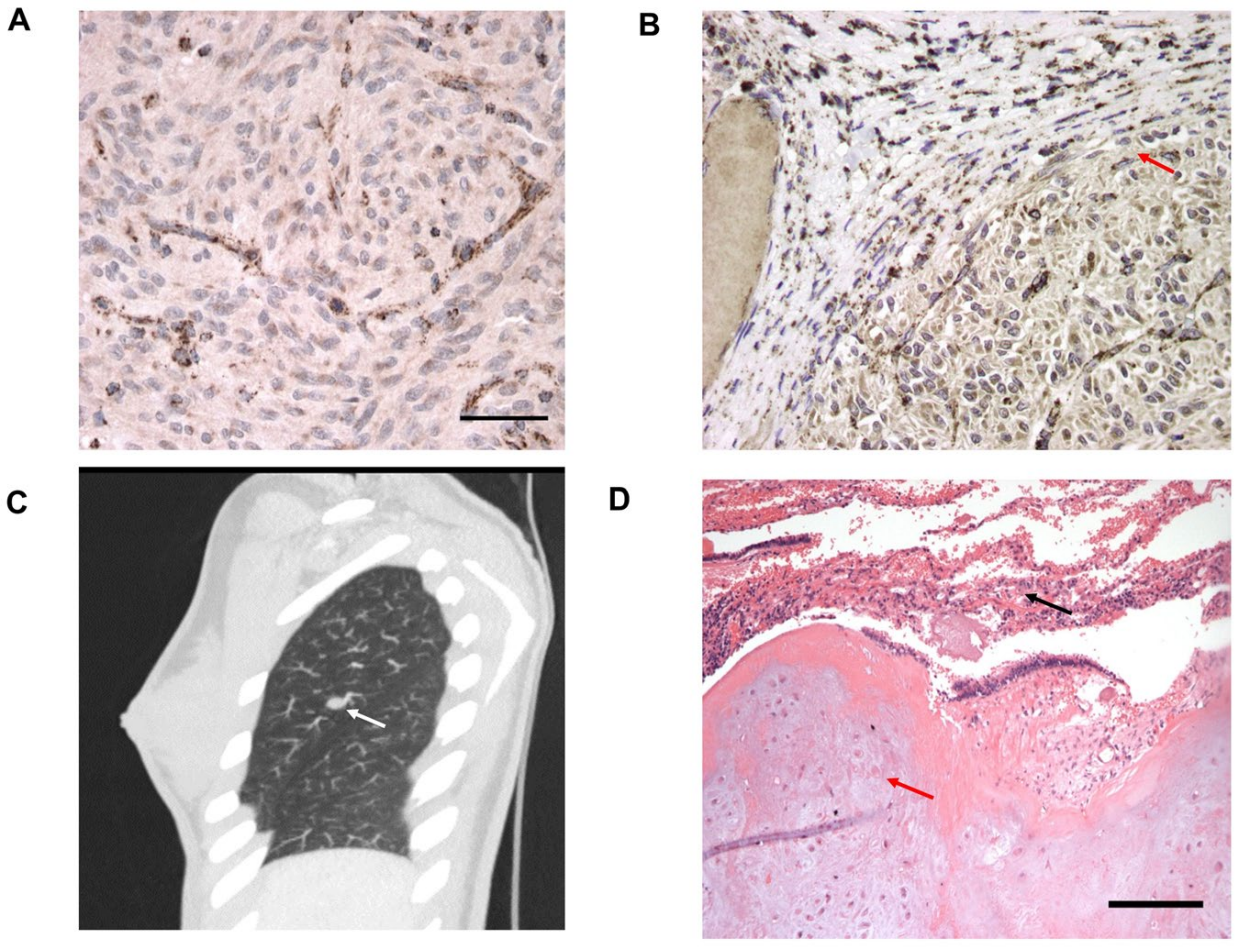
Figure (**A**) and (**B**) shows loss of SDHB protein expression on immunohistochemical analysis of the primary wtGIST tumour in case #001 and #003 respectively. In Figure (**B**) SDHB expression is preserved in adjacent normal tissue as highlighted by the red arrow. Figure (**C**) shows a pulmonary chondroma in case #021 as demonstrated by the white arrow and Figure (**D**) demonstrates the histology of a pulmonary chondroma from case #004, with evidence of normal collapsed lung tissue illustrated by the black arrow and chondrocytes in the tumor marked by the red arrow.

Tumour sequencing was performed on 4/6 (#001, #002, #003, #004) cases with evidence of *SDHC* hypermethylation and no somatic *SDHx* variants were detected. SDHB immunohistochemistry was performed on all tumours and loss of SDHB expression was confirmed in all 6 cases with *SDHC* hypermethylation (Table 1, examples for #001 and #003 displayed in Fig. 3A,B).

### Data extracted from whole genome germline sequencing analysis (WGS) of hypermethylated cases

WGS data was analysed for three cases with tumour *SDHC* hypermethylation for whom sufficient DNA was available (cases; #002, #021 and #022). No candidate pathogenic structural or single nucleotide variants were identified in these three cases in the *SDHC* locus (between 161314257-161375340) containing the SDHC promoter, exons and 3′UTR. In the absence of an *in cis* genetic cause, additional analysis for potential pathogenic variants in genes implicated in genome methylation (*TET1*, *TET2*, *TET3*, *DNMT3B*, *DNMT3A*, *DNMT1*), was performed.

10/965 filtered variants (in test and control samples) were detected in 3 genes (Table S2). A comparison of the identified variant frequencies in the three *SDHC* hypermethylation samples compared to 4053 control genomes with low neoplastic risk (from the NIHR Rare Diseases BioResource BRIDGE project) did not yield any statistically significant findings (Benjamini Hochberg correction for a false discovery rate of p values was applied and based on 965 tested hypotheses).

None of the variants identified in the *SDHC* methylation cases were considered to be pathogenic by ACMG criteria. A missense variant of uncertain significance in *TET2* (p.Ile1762Val) was identified in all three cases with *SDHC* promoter hypermethylation, but this variant was absent from 1000 genomes and UK10K databases and was identified in 1876/4053 controls (Table S2).

### Investigating SDHC hypermethylation in non PPGL and wtGIST tumour sets

To further investigate the apparent specificity of *SDHC* epimutations in dSDH wtGIST we explored whether *SDHC* epimutations might occur in non-wtGIST tumours with (a) DNA hypermethylation or (b) low *SDHC* expression in order to test the hypothesis that an *SDHC* epimutation is specific to particular tumour types and/or is not a consequence of generalised tumour DNA hypermethylation.

Firstly we undertook *SDHC* promoter methylation analysis on 17 *IDH1* mutant glioma samples. *IDH1* mutant gliomas have previously been associated with a global hypermethylation phenotype due to inhibition of alpha ketoglutarate dependent de-methylating enzymes^20^. The mean *SDHC* promoter methylation in the *IDH1* mutant glioma samples was 2% (±SD 1.28, range 1–4%) (Fig. 1A and Table S3).

Secondly, from non-wtGIST tumours with *SDHC* gene expression data and sequencing data from cancer genomic studies (accessed at http://www.cbioportal.org/), we identified 25 tumour samples with very low *SDHC* transcript levels and no *SDHC* mutation (Table S4). Methylation array (Illumina 450k) data for these 25 tumours was accessed and beta values for 13 *SDHC* promoter probes inspected. None of the tumours showed evidence of *SDHC* promoter hypermethylation (Table S4).

## Discussion

A search of PubMed (using the terms *SDHC* and methylation or epimutation) identified 8 publications containing 34 cases of *SDHC* promoter region hypermethylation in a variety of tumour types including dSDH wtGIST, sympathetic (PGL) and parasympathetic (HNPGL) paragangliomas^1,9,10,12,13,18^ (Table S5). The majority of patients (94%, 32/34) identified with *SDHC* hypermethylation had a dSDH-wtGIST and 53% (18/34) of these cases also had an additional tumour(s) (Table S5).

### Phenotype of *SDHC* epimutation cases detected in the present study

We identified *SDHC* promoter region methylation in 6/15 wtGIST (all 6 cases were dSDH-wtGIST) but none of the 17 PPGL or SDH-preserved-wtGIST (3/15 wtGIST). All *SDHC* hypermethylation cases were female and were significantly younger than patients without an *SDHC* epimutation.

Combining our results with previously published series (see Table S5), the association with dSDH-wtGIST (alone or as the presenting feature of a multi-tumour syndrome), female gender and young age at diagnosis is maintained. Rare reports of isolated sympathetic and parasympathetic PGL with an *SDHC* epimutation have also been published (Table S5).

In two of the cases reported here, somatic *SDHC* promoter methylation was detected in the presence of a germline pathogenic *SDHC* variant. This would be consistent (though not proven) with a two hit model of tumourigenesis in which *SDHC* hypermethylation resulted in silencing of the wild-type allele in the tumour. Two of the cases with a germline *SDHC* mutation had multiple tumours including case #004 (Fig. 3C,D). The association of synchronous or metachronous gastric wtGIST, PPGL and pulmonary chondroma (PCHO) is referred to as Carney triad whereas the combination of GIST and PPGL is designated as the Carney-Stratakis syndrome (CSS) or dyad. Although it was previously suggested that PCHO occurred exclusively in CT (a non-inherited disorder), this study and others^11,19^ have demonstrated that the triad of wtGIST, PPGL and PCHO can occur in association with a germline *SDHx* mutation and highlights the overlapping features of CT and CSS^19–21^. However, we did not (from interrogation of TCGA, literature and original data) find evidence that *SDHC* promoter methylation occurs outside of wtGIST and, occasionally, PGL.

We identified 4 cases of tumour *SDHC* promoter methylation with no detectable germline or somatic *SDHC* mutations. Furthermore there was no evidence of a germline *SDHC* epimutation. In such cases the *SDHC* promoter hypermethylation might be a somatic event as occurs in many types of cancer and multiple tumour suppressor genes^22^. In the case of the mismatch repair gene *MLH1*, somatic *MLH1* promoter methylation is relatively common in older individuals with colorectal cancer with microsatellite instability but there are rare cases of patients with a constitutional *MLH1* epimutation who present at a younger age^23^. In contrast to *MLH1*, there has been no evidence to date that *SDHC* epimutations may result from *in cis* promoter region genetic variants^24^, although some studies have described mosaic constitutional *SDHC* promoter hypermethylation in association with tumour hypermethylation^8^. In the absence of a detectable *in cis* or *in trans* genetic variant in these cases, low level postzygotic tissue mosaicism for *SDHC* promoter hypermethylation, provides an alternative hypothesis for this multiple tumour phenotype at a young age.

### Translating the diagnosis of an *SDHC* epimutation into clinical practice

A primary aim of this study was to develop a proposed methodology for diagnostic *SDHC* promoter methylation testing in a clinical setting. We developed a pyrosequencing-based method because it is well established on FFPE material, allows a low level variant detection and is frequently used in diagnostic pathology services for other types of somatic methylation analysis (e.g. *MGMT* promoter methylation analysis in glioma). Our method worked well on DNA extracted from archived routine diagnostic FFPE material (an important consideration as fresh frozen tumour is rarely available) and pyrosequencing is less expensive compared to alternative methods e.g. methylation arrays.

We found that the methylation status of 12 CpG’s in CpG27 in the promoter region of the *SDHC* gene could be accurately assessed and that detection of hypermethylation of the *SDHC* promoter correlated with reduced *SDHC* mRNA on mRNA extracted from the same FFPE tissue block. Recently described methods for the detection of *ex-vivo* and *in vivo* succinate accumulation are useful adjuncts to SDHB IH for the detection of SDH deficiency^25,26^. However, these methods cannot identify the cause of SDH deficiency. The authors recommend that whenever possible, cases with *SDHC* promoter hypermethylation should be analysed by RT-PCR of both tumour and adjacent normal tissue to confirm silencing of *SDHC* in the tumour tissue.

**Figure 4:**
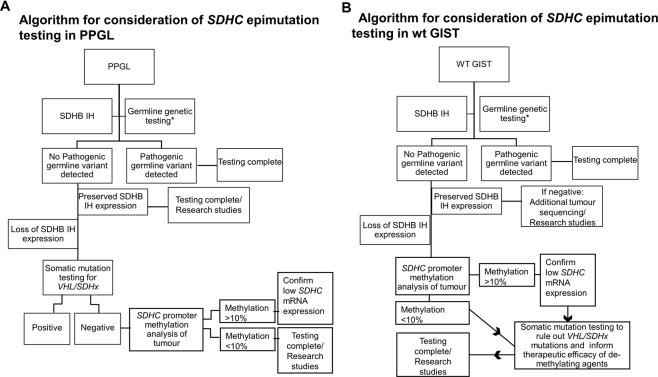
Illustrates a proposed work flow for the investigation of *SDHC* promoter methylation in a clinical setting for (**A**) PPGL and (**B**) wtGIST (defined as a GIST with no identified somatic mutation in *KIT*, *PDGFRA* OR *BRAF*) *Next generation sequencing panel for PPGL including the genes; *SDHA*, *SDHB*, *SDHC*, *SDHD*, *SDHAF2*, *FH*, *TMEM127*, *RET*, *VHL*, *MAX* and including multiplex ligation dependent probe amplification for deletions and duplication. **Next generation sequencing panel for wtGIST including the genes; *SDHA*, *SDHB*, *SDHC*, *SDHD*, *KIT, PDGFRA, NF1* and including multiplex ligation dependent probe amplification for deletions and duplication.

Given that SDHB immunohistochemistry is a relatively accessible and sensitive test, this should be considered as a first-line triaging test for the detection of SDH deficiency in PPGL and wtGIST^21^. We recommend that germline genetic testing is always considered as the next diagnostic step in dSDH tumours to rule out a potential syndromic cause. If germline genetic testing (including MLPA) is negative and SDHB IH suggests loss of SDHB protein expression, the first step for PPGL should be somatic sequencing^27^ to investigate for somatic *SDHx* or *VHL* mutations, which can account for loss of SDHB protein expression^6^. However, as *SDHC* epimutations are more frequent in wtGIST than in PPGL, we recommend *SDHC* promoter methylation analysis as the next step after germline genetic testing for wtGIST (Fig. 4B). If an *SDHC* epimutation is diagnosed, somatic tumour sequencing should be performed to identify a co-existing somatic *SDHx* mutation, which may affect the efficacy of any potential demethylating therapy (Fig. 4).

Importantly, a number of potential limitations in the diagnosis of *SDHC* methylation using pyrosequencing methods on FFPE tumour tissue, were encountered over the course of this study. Identification of these pitfalls has prompted the following practical recommendations; i) using a minimum input of 50 ng of bisulfite converted DNA for the PCR and ii) a minimum volume of 10 microlitre of the PCR product for pyrosequencing can minimize the risk of false elevations in methylation, iii) fully methylated and unmethylated human control DNA, treated with bisulfite should be used as external controls on each pyrosequencing run and iv) the use of matched normal tissue is useful as an internal control to account for any false elevation in methylation which may have been caused by the long term paraffin storage. Limitations of this study also include the retrospective study design and relatively small sample size and diagnostic laboratories wishing to adopt the methodology described herein will need to undertake a formal clinical validation study before implementing it for clinical diagnostic use.

In conclusion, the results from our literature review, experimental studies and interrogation of the TCGA data, suggest that *SDHC* epimutations are rare in tumours other than wtGIST and PPGL. Improving the accessibility of clinical diagnostic testing for *SDHC* promoter methylation will facilitate the management of patients with wtGIST by enabling stratification for personalised therapeutic strategies and defining risks for other family members, according to the presence or absence of a germline *SDHx* mutation and or a *SDHC* epimutation.

## Supplementary information

Dataset 1

## Data Availability

Data is provided in the manuscript and/or Supplementary Data.
