# Supplementary material for: SDHC epi-mutation testing in gastrointestinal stromal tumours and related tumours in clinical practice
Source: Sci Rep. 2019 Jul 15;9:10244. doi: 10.1038/s41598-019-46124-9 (PMC6629852; doi:10.1038/s41598-019-46124-9)
Supplement: Supplementary file 1 — Dataset 1 [file 41598_2019_46124_MOESM1_ESM.pdf]

SDHC epi-mutation testing in gastrointestinal stromal tumours and related tumours in clinical practice

Ruth T. Casey[1, 2], Rogier ten Hoopen[3], Eguzkine Ochoa[1], Benjamin G. Challis[1, 2], James Whitworth[1], Philip S Smith[1], Jose Ezequiel Martin[1], Graeme R Clark[1], Fay Rodger[1], Mel Maranian[1], Kieren Allinson[4], Madhu Basetti[5], Thomas Roberts[6], Luis Campos[6], Joanne Anstee[4], Soo-Mi Park[1], Alison Marker[4], Colin Watts[7],Venkata R Bulusu[8], Olivier T Giger[4,9*], Eamonn R Maher[1*]

1. Department of Medical Genetics, University of Cambridge and NIHR Cambridge Biomedical Research Centre and Cancer Research UK Cambridge Centre, CB2 OQQ, United Kingdom.

2. Department of Endocrinology, Cambridge University NHS Foundation Trust, Cambridge, CB2 OQQ, United Kingdom.

3. Department of Oncology, University of Cambridge, Addenbrooke's Hospital, Cambridge, CB2 0QQ, UK

4. Department of Histopathology Cambridge University NHS Foundation Trust and Cancer Research UK Cambridge Centre Cambridge, CB2 0QQ, United Kingdom.

5. Cancer Research UK Cambridge Institute, University of Cambridge, Li Ka Shing Centre, Robinson Way, Cambridge CB2 0RE, UK.

6. Haematology Oncology Diagnostic Service (HODS), Cambridge University NHS Foundation Trust, Cambridge, CB2 OQQ, United Kingdom.

7. Institute of Cancer and Genomic Sciences, College of Medical and Dental Sciences, University of Birmingham, Birmingham B15 2TT, UK

8. Department of Medical Oncology, Cambridge University NHS Foundation Trust, Cambridge, CB2 OQQ, United Kingdom.

9. Department of Pathology, University of Cambridge, Addenbrooke's Hospital, Cambridge, CB2 0QQ, UK.

*Joint senior authors

Corresponding author: Dr Ruth Casey, Department of Medical Genetics, University of Cambridge and NIHR Cambridge Biomedical Research Centre and Cancer Research UK Cambridge Centre, CB2 OQQ, United Kingdom.

Email: rc674@medschl.cam.ac.uk

Supplementary data:

1. Methods

i) Tumour DNA extraction

Tissue slides were de-paraffinised by two soakings for 10 min in 100% xylene, and two soakings for 10 min in 100% ethanol and then air-dried. The tissue was scraped with a sterile blade and suspended in 180 ml of digestion buffer ATL (QiaAmp MiniElute). 20 microliter proteinase K was added and the tissue was incubated for 3 days at 56°C with a daily 10 microliter proteinase K supplement. Subsequently, the digested tissue was incubated for one hour at 90°C, centrifuged, supplemented with 200 microliter of AL buffer, mixed by vortexing, supplemented with 200 microliter of 100% ethanol and mixed by vortexing and "briefly centrifuged. The lysate was loaded on a QiaAmp MinElute Column and centrifuged 10000 rcf for 1 minute, washed with 500 microliter of AW1, centrifuged at 10000 rcf for 1 minute, washed with 500 microliter AW2 and centrifuged at 16000 rcf for 3 min, then centrifuged dry for 3 min at 16000 rcf, and eluted with 50 microliter ATE buffer by incubation at room temperature for 5 min and centrifugation for 2 min at 10000 rcf.

ii) Analysis of TCGA tumour set

The c-bioportal (http://www.cbioportal.org) dataset was interrogated for tumours with *SDHC* expression below the median expression level for the given tumour type and without a sequence mutation. Thirty tumours were identified and 450k infinum methylation array data was available for 25/30 tumours from the cancer genome atlas (TCGA)

(https://cancergenome.nih.gov). Open access data was downloaded for the 25 tumours and analysed for evidence of *SDHC* promoter methylation based on the β value across *SDHC* promoter CpG targets mapping to the TSS-proximal CGI. The results published herein are therefore in part based upon data generated by The Cancer Genome Atlas managed by the NCI and NHGRI.

iii) RNA extraction

For RNA extraction slides were processed with the RNAstorm kit from CellDataSci (CD501) according to the manufacturer's protocol. After histological identification and selection of the tissue region to be processed for RNA extraction the relevant tissue is scraped off using a sterile scalpel blade (Swann Morton No.11) into 500 microliter of de-paraffinisation reagent, vortexed for 10 sec and incubated for 3 min at 72°C, then allowed to cool to room temperature. 80 microliter of CAT5 reagent was added, vortexed for 10 sec and centrifuged for 1 min at 16000 rcf. Most of the clear yellow organic upper phase was removed and discarded, the remainder incubated for 30min at 72°C, followed by adding 80 microliter lysis buffer and 10 microliter of protease K and continued incubation at 72°C for 2 hours. The RNA extraction was cooled for 3 min on ice and centrifuged for 15 min at 16000 rcf. The lower aqueous phase was removed to a new microcentrifuge container, supplemented with 150 microliter binding buffer, 450 microliter 100% Ethanol and mixed by inverting several times and loaded on a spin column. The spin column was centrifuged for 1 min at 16000 rcf and washed with 300 microliter of DNase treatment wash buffer (1:1:3 of water, binding buffer and ethanol, respectively) and centrifuged for 1 min at 16000 rcf. 70 microliter Dnase I buffer with 2 microliter reconstituted DNase I was added to the spin column membrane, incubated for 15 min at RT and centrifuged for 1 min at 16000 rcf. Subsequently the spin

column was washed with 300 microliter of DNAse treatment wash buffer and centrifuged for 1 min at 16000 rcf, 500 microliter Wash buffer, centrifuged for 1 min at 16000 rcf, and extra wash of 500 microliter Wash buffer, centrifuged for 1 min at 16000 rcf, and spun dry for 5 min at 16000 rcf. RNA was eluted from the spin column with 30 microliter RNAse-free water for 1 min incubation at RT and centrifugation for 2 min at 10000 rcf. RNA yield was measured on the Nanodrop ND-1000 Spectrophotometer and stored at minus 80°C.

iv) cDNA Synthesis

cDNA synthesis was performed with the Superscript III First –Strand Synthesis Supermix (Invitrogen 18080-400) and according to the manufacturers protocol.

v) Expression Analysis with Quantitative RT PCR

Relative expression analysis was performed with a predesigned Taqman Gene Expression assay for *SDHC* (SDHC: Hs01698067_s1) (Applied Biosystems, Hs01698067_s1, FAM) and using GAPDH as a reference gene (Applied Biosystems, Hs02758991_g1, FAM). Each sample was run in triplicates on Applied Biosystems Life Technologies QuantStudio 6Flex optical thermal cycler. The PCR conditions were 10 min at 95˚C primary activation/denaturation step, followed by 45 cycles of a 15sec 95˚C denaturation and a 1min 60˚C annealing/elongation step at which time the fluorescence of the Taqman probe is measured.

vi) Statistical Analysis

R studio (1.1.447) was employed for analysis of differential *SDHC* mRNA expression and for methylation analysis and visualisation. Statistical analysis was performed using MedCalc (version 18.2.1). A mean and standard deviation was calculated for all continuous variables. An unpaired student t-test was employed to investigate differences between groups.

ROC curve analysis was performed to determine the optimal mean methylation cut off to differentiate *SDHC* epimutant cases from non epimutant cases. A sample size of 40 was required (10 positive cases and 31 negative cases) to ensure a minimum type 1 and type 2 error of 0.2. Therefore to achieve this sample size data from this study was combined with the data published by Haller et all (9) (including four cases with CT and five negative control samples) for ROC curve analysis.

Table S1: % methylation at each of the 12 CpG's of CpG27 in the SDHC promoter region in PPGL and wtGIST samples

Case	CpG1	CpG2	CpG3	CpG4	CpG5	CpG6	CpG7	CpG8	CpG9	CpG10	CpG11	CpG12
**001**	53	80	73	75	74	71	75	69	73	77	83	75
**002**	20	34	31	41	52	48	52	46	46	54	63	51
**003**	63	69	68	68	68	64	66	65	66	72	79	62
**004**	43	48	46	45	48	47	43	38	41	50	50	44
**005**	3	5	4	4	8	6	5	6	6	7	7	6
**006**	1	1	1	1	1	1	0	0	1	1	0	1
**007**	1	1	0	1	8	1	1	1	3	3	2	2
**008**	0	1	1	2	3	3	1	3	1	2	3	2
**009**	0	2	1	1	1	1	1	1	1	2	2	1
**010**	0	2	1	1	1	1	1	1	1	2	2	1
**011**	0	1	0	0	1	1	0	0	1	1	1	2
**012**	2	0	0	0	2	1	0	0	1	1	1	2
**013**	0	1	1	1	1	1	1	1	1	1	2	2
**014**	0	1	0	0	1	1	1	1	1	3	3	2
**015**	1	1	1	1	2	1	0	1	2	2	3	1
**016**	1	1	1	1	2	2	1	2	2	2	3	3
**017**	7	5	3	4	10	8	5	5	4	9	9	7
**018**	5	6	5	5	7	6	4	5	5	6	6	6
**019**	2	2	2	3	5	4	5	3	5	5	6	5
**020**	0	1	1	1	1	1	1	0	1	1	2	1
**021**	33	39	45	47	59	50	54	45	44	59	66	52
**022**	27	33	28	34	40	33	36	31	28	32	35	31
**023**	4	7	6	8	10	10	10	8	7	10	10	9
**024**	2	2	2	3	5	4	5	3	5	5	6	5
**025**	0	2	2	0	2	2	0	0	1	1	0	3
**026**	1	1	1	1	2	1	0	1	2	2	3	1
**027**	0	1	0	0	2	1	0	0	1	1	1	1
**028**	1	1	1	2	5	5	4	2	2	5	4	3
**029**	0	0	0	0	1	1	1	1	1	1	3	3
**030**	0	1	1	0	1	1	1	1	1	1	2	2
**031**	0	0	0	0	1	1	0	0	1	8	1	1
**032**	0	1	1	0	3	4	3	5	5	5	8	6

Table S2: Table of variants identified in genes associated with regulation of methylation on whole genome sequencing analysis

RS ID	Gene	Protein change	EXAC	Number of cases	Control	Pathogenicity
rs61741171	*TET3*	p.(Pro294Ser)	0.04	1	441	Uncertain significance
rs6843141	*TET2*	(p.Val218Met)	0.05	1	133	Uncertain significance
rs17253672	*TET2*	(p.Pro363Leu)	0.04	1	413	Uncertain significance
rs34402524	*TET2*	(p.Leu1721Trp)	0.1	1	881	Uncertain significance
rs2454206	*TET2*	(p.Ile1762Val)	0.3	3	1876	Uncertain significance
rs10823229	*TET1*	(p.Asp162Gly)	0.3	1	1872	Uncertain significance
rs12773594	*TET1*	(p.Ser193Thr)	0.1	1	1001	Uncertain significance
rs12221107	*TET1*	(p.Ala256Val)	0.1	1	523	Uncertain significance
rs16925541	*TET1*	(p.Asn1018Ser)	0.1	1	488	Uncertain significance
rs199882600	*TET1*	(p.Val2128Ile)	.0004	1	2	Uncertain significance

Table S3: % methylation at each of the 12 CpG's in the *IDH1* mutant glioma samples

Case	CpG1	CpG2	CpG3	CpG4	CpG5	CpG6	CpG7	CpG8	CpG9	CpG10	CpG11	CpG12
**GL1**	1	1	1	2	1	1	2	2	2	2	2	
**GL2**	2	2	2	3	5	5	5	3	4	5	5	4
**GL3**	1	1	1	2	3	3	3	3	3	3	3	3
**GL4**	1	0	0	0	1	1	0	1	1	1	2	2
**GL5**	1	1	1	1	1	1	1	1	1	1	2	2
**GL6**	1	1	1	1	1	1	1	1	1	2	2	
**GL7**	1	1	1	1	2	1	1	1	2	2	2	2
**GL8**	1	1	1	1	2	2	1	1	2	1	2	2
**GL9**	1	1	0	0	1	1	1	1	1	1	1	1
**GL10**	1	1	1	1	1	1	1	1	1	2	2	2
**GL11**	1	1	1	0	1	1	1	1	1	1	2	1
**GL12**	3	3	3	3	3	3	3	3	4	3	5	3
**GL13**	1	1	1	0	1	1	1	1	1	1	1	1
**GL14**	2	2	2	2	3	3	3	3	3	3	4	3
**GL15**	4	4	4	5	7	6	7	5	7	6	7	6
**GL16**	2	2	2	2	3	3	4	3	3	3	4	3
**GL17**	0	1	0	0	1	1	1	0	1	1	1	1

Table S4: TCGA tumour set with differential *SDHC* expression analysed for *SDHC* promoter hypermethylation

TCGA ID	Tumour type	Mean β-value across *SDHC* promoter
TCGA-5P-A9K2	Papillary RCC	0.06
TCGA-G7-A8LB	Papillary RCC	0.04
TCGA-B1-A47N	Papillary RCC	0.05
TCGA-P4-A5EA	Papillary RCC	0.13
TCGA-P4-A5ED	Papillary RCC	0.05
TCGA-DX-A2JO	Sarcoma	0.06
TCGA-DX-AB2V	Sarcoma	0.06
TCGA-DX-A48N	Sarcoma	0.05
TCGA-QR-A708	PPGL	0.06
TCGA-KL-8327	Chromophobe RCC	0.06
TCGA-46-6026	Squamous cell Lung carcinoma	0.06
TCGA-BO-4811	Clear cell RCC	0.05
TCGA-CF-A3MF	Bladder Cancer	0.05
TCGA-VD-AA80	Uveal melanoma	0.06
TCGA-OR-A5JX	Adrenocortical carcinoma	0.05
TCGA-BS-A0V7	Uterine Cancer	0.06
TCGA-ZM-AA05	Testicular germ cell tumour	0.06
TCGA-ZM-AA06	Testicular germ cell tumour	0.04
TCGA-HC-7752	Prostate Cancer	0.05
TCGA-KK-A59V	Prostate Cancer	0.05
TCGA-XU-AAXZ	Thymoma	0.06
TCGA-EM-A2CR	Thyroid Cancer	0.05
TCGA-EM-A4FR	Thyroid Cancer	0.05
TCGA-EM-A22Q	Thyroid Cancer	0.05
TCGA-AB-2952	AML	0.06

Table S5: Reported cases of tumours with evidence of *SDHC* promoter methylation

*=450K Infinium methylation beadarray [Gene Expression Omnibus (GEO) platform GPL13534, **=methylation-sensitive high-resolution melting (MS-HRM), ***= some cases reported in Boikos et al are believed to be originally reported in reference (6)

**Tumour phenotype**	**Age at presentation**	**Sex**	**Co-existing germline *SDHx* mutation**	**Method employed for methylation analysis**	**Reference**
wt GIST+PGL+PCHO	8	F	No	450k array*	8
wt GIST	8	F	No	450k array*	8
wt GIST	9	F	No	450k array*	8
wt GIST	10	F	No	450k array*	8
wt GIST	11	F	No	450k array*	8
wt GIST	12	F	No	450k array*	8
wt GIST	13	F	No	450k array*	8
wt GIST+PGL+PCHO	14	F	No	450k array*	8
wt GIST	15	F	No	450k array*	8
wt GIST+PGL+PCHO	18	F	No	450k array*	8
wt GIST+PGL+PCHO	22	F	No	450k array*	8
wt GIST	11	F	No	450k array*	8
wt GIST	18	F	No	450k array*	8
wt GIST	19	F	No	450k array*	8
wt GIST	57	F	No	450k array*	8
wt GIST+PGL+PCHO	26	M	*SDHC*	450k array*	8
wt GIST	28	M	*SDHC*	450k array*	8
wt GIST+PGL+PCHO	28	M	*SDHC*	450k array*	8
wt GIST	47	F	*SDHC*	450k array*	8
wt GIST +PCHO	37	M	*No*	450k array*	11
wt GIST+PCHO	28	F	*No*	450k array*	11
wt GIST+PCHO	26	F	*No*	450k array*	11
wt GIST+PCHO	14	F	*No*	450k array*	11
wt GIST+PCHO	18	F	*No*	450k array*	11
wt GIST+PGL	18	F	*No*	450k array*	11
wt GIST+PGL+PCHO	22	F	*No*	450k array*	11
wt GIST+PGL+PCHO	25	F	No	Massive parallel bisulfite sequencing	9
wt GIST+PGL+PCHO	34	F	No	Massive parallel bisulfite sequencing	9
wt GIST+PGL+PCHO	11	F	No	Massive parallel bisulfite sequencing	9
wt GIST+PGL	15	F	No	Massive parallel bisulfite sequencing	9
wt GIST	25	F	No	Methylation assay	10
PGL	25	M	No	450k array*	12
Multifocal PGL	33	F	No	(MS-HRM) analysis and Sanger-sequencing of the *SDHC* promotor region**	13
wtGIST+ PGL	NA	F	No	450k array*	19

Table S6: Demographic data of Glioma cases included in this study

Case	Gender	Age
**GL1**	F	39
**GL2**	M	22
**GL3**	M	35
**GL4**	M	47
**GL5**	M	30
**GL6**	F	34
**GL7**	F	48
**GL8**	M	25
**GL9**	F	49
**GL10**	F	31
**GL11**	F	40
**GL12**	M	45
**GL13**	M	44
**GL14**	M	41
**GL15**	M	71
**GL16**	M	28
**GL17**	**F**	**27**

Figure S1: This histogram demonstrates the fold difference of *SDHC* mRNA expression (corrected for GAPDH) in tumour compared to adjacent normal tissue. Those bars represented in grey show a negative fold difference in tumour compared to normal tissue and black bars represent a positive fold difference in tumour compared to normal tissue. Though most cases without *SDHC* hypermethylation showed higher levels of *SDHC* expression in the tumour than in the adjacent normal tissue (23/26), three tumours showed reduced expression. The lowest SDHC expression level was noted in case #0026, which had a -15-fold difference. This case was a phaeochromocytoma with a known pathogenic germline *VHL* variant (c.499C>G, p.Arg167Gly) and showed an equivocal result on SDHB immunohistochemistry testing, but no evidence of a somatic *SDHx* mutation (See Figure S1). In the absence of a somatic or germline *SDHx* mutation or an *SDHC* epimutation, these findings could be explained by the oxidoreductase defect associated with *VHL* mutations (1).

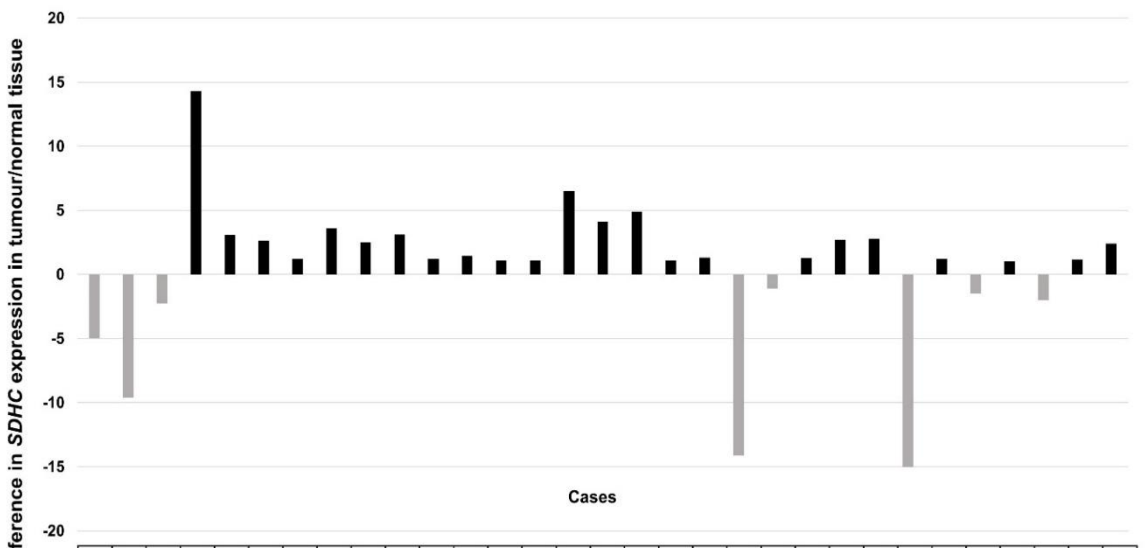

2	3	4	5	6	7	8	9	10	11	12	13	14	15	16	17	18	19	20	21	22	23	24	25	26	27	28	29	30	31	32
-5	-9.61	-2.26	14.3	3.1	2.65	1.2	3.6	2.5	3.11	1.2	1.46	1.1	1.1	6.5	4.1	4.9	1.1	1.3	-14.1	-1.1	1.28	2.7	2.8	-15	1.2	-1.5	1	-2	1.17	2.4
